# Acute Effects of Flywheel Eccentric Overload on Vertical Jump, Change of Direction, and Isometric Mid-Thigh Pull Performance in Top-Level Team Sports Athletes

**DOI:** 10.3390/sports14010006

**Published:** 2026-01-01

**Authors:** Nikola Andrić, Tatjana Jezdimirović-Stojanović, Mladen Mikić, Bojan Međedović, Damjan Jakšić, Marko D. M. Stojanović

**Affiliations:** 1 Faculty of Sport and Physical Education, University of Novi Sad, 21000 Novi Sad, Serbia; nikola.trenaznaekspertiza18@gmail.com (N.A.); mladen.mikic@fsfvns.edu.rs (M.M.); damjan.jaksic@fsfvns.edu.rs (D.J.); 2 Training Expertise, 21000 Novi Sad, Serbia; 3 Faculty of Sport, Union-Nikola Tesla University, 11000 Belgrade, Serbia

**Keywords:** post-activation potentiation, complex training, team sports, sports performance

## Abstract

We examined the acute effects of flywheel eccentric overload (FEO) on countermovement jumps (CMJs), changes of direction (COD), and isometric mid-thigh pulls (IMTPs) in top-level team sports athletes (three females and seven males). FEO was carried out by performing 3 × 6 reps with 0.025 kg·m^2^ inertia and a 2 min passive rest period. Its post-activation potentiation was compared to a control warm-up. Performance was tested at 0, 3, and 6 min post-intervention. Significant improvements were reported in the COD5m times for the left (F = 8.38, *p* < 0.001, ES = 1.92) and right legs (F = 11.3, *p* < 0.001, ES = 2.24), as well as for CMJ height (F = 12.4, *p* < 0.001, ES = 2.35). Significant differences were observed in COD5m between baseline and 3 min (*p* < 0.001, ES = 0.99 and *p* = 0.003, ES = 1.25) and 6 min (*p* = 0.04, ES = 1.19 and *p* < 0.001, ES = 1.09) for the left and right legs, respectively. Jump height increased significantly at 3 min (*p* < 0.001, ES = 1.62) and remained elevated at 6 min (*p* < 0.001, ES = 1.02). CMJ peak power (CMJPP) decreased significantly (F = 6.4, *p* = 0.002, ES = 1.68), with a drop at 0 min (*p* = 0.024, ES = 0.85) and a return to baseline at 3 min (*p* = 0.002, ES = 1.35). No significant effects were found for the CMJ eccentric rate of force development (CMJRFDecc) or IMTP. It was found that FEO can acutely enhance jumping and changes of direction but not strength in elite team sports athletes. A three-minute rest appears to maximize these effects.

## 1. Introduction

Post-activation performance enhancement (PAPE) describes the increase in muscle strength or power output that occurs following previous maximal or near-maximal muscle contractions [1]. Although the exact physiological mechanisms of this phenomenon remain to be elucidated [1,2], central and peripheral mechanisms are most frequently outlined. Concisely, central mechanisms relate to increased (1) recruitment of higher-order motor units, (2) conduction velocity of muscle fibers, and (3) motor neuron excitability, while peripheral mechanisms relate to the phosphorylation of the myosin regulatory light chain and increased calcium (Ca^2+^) sensitivity. These acute physiological changes increase the rate and magnitude of force generated by the myofilaments and consequently improve athletic performance [3].

The practical application of PAPE primarily involves the use of complex training in which practitioners combine maximal or near-maximal muscle contractions (i.e., conditioning activities) with strength/power exercises (e.g., back squats and countermovement jumps) to create strength–power potentiation complexes [4]. The objective is to facilitate the transfer of maximal strength into enhanced strength/power development [5]. Although several studies have shown that complex training can acutely enhance strength [6], power [7], and change-of-direction performance [8], conflicting results have been reported regarding the extent of improvement, with increases, no changes, and decreases all having been reported [2,9]. A thorough review of existing research indicates that acute enhancements in muscular performance following a strength–power potentiation protocol are influenced by the delicate interplay between fatigue and potentiation, which coexist to varying extents after engagement in a conditioning activity [10]. Muscle performance improves when potentiation outweighs fatigue, remains stable when both are balanced, and declines if fatigue is excessive. In addition, this equilibrium appears to be affected by both individual characteristics and the specific nature of the strength–power potentiation process [2]. Specifically, evidence suggests that individuals with greater strength can demonstrate larger and more rapid potentiation responses compared to weaker individuals [11]. When considering the strength–power potentiation complex, it seems that the interaction between potentiation and fatigue is probably linked to the type of muscle contraction performed during the conditioning activity [12].

Early studies on this subject primarily focused on either isometric contractions (such as sustained or intermittent maximal voluntary efforts) or dynamic conditioning activities, including loaded jumps, sprints, throwing exercises, and resistance training [3]. Isometric activities tend to generate more central fatigue and likely activate the peripheral mechanisms of PAPE, whereas dynamic activities seem to have the opposite effect [2]. Consequently, each type of potentiation may operate through distinct pathways, influencing the timing, extent, and duration of the PAPE effects [13]. Multiple studies have explored the impact of isometric MVCs on subsequent explosive actions; while a few reported performance improvements [14,15], others showed no observable change [16,17]. Similarly, some research has employed dynamic maximal or near-maximal voluntary contractions to trigger PAPE, with certain findings indicating enhanced performance afterwards [18,19,20], while other studies found no significant effects [21,22]. Given the different physiological factors associated with PAPE across various contraction types and the inconsistent results from studies on isometric and dynamic training methods, it appears necessary to investigate alternative training approaches to elicit PAPE effects. In this context, recent findings have highlighted the potential of flywheel-based exercises as viable alternative protocols for inducing PAPE [12,23]. Flywheel training, introduced in the early 1990s, relies on the energy generated during a maximal concentric effort, which is then preserved and utilized during the following eccentric phase owing to the system’s inertia, resulting in an intensified eccentric load when a brief, focused braking movement occurs [24]. This unique resistance training approach enables maximal force production across the entire range of motion, featuring phases where eccentric force demands are greater than concentric ones [25]. The portability of these devices allows practitioners to use them in real sport settings, further increasing their practical applications. The well-established physiological advantages of eccentric contractions (enhanced motor unit recruitment) may relate to central mechanisms underlying PAPE [2]. In addition, enhanced eccentric force production can boost the performance of the stretch–shortening cycle, potentially leading to more pronounced transfer effects on rapid, integrated eccentric–concentric movements involved in performance outcomes such as changing direction and jumping [26]. Collectively, these factors could play a role in refining the balance between training-induced fitness gains and fatigue management [27] and consequently enhance PAPE.

A number of studies examined the acute effects of flywheel eccentric overload (FEO) on team-sport-specific performance outcomes. As summarized by Beato et al. [12], FEO, conducted with single or multiple sets, at various intensities (0.03–0.11 kg·m^2^), and with short rest period intervals (3–9 min), seems to be effective in enhancing squat jump, squat jump power [28], countermovement jump, and change of direction ability [29,30,31], as well as knee flexion and extension strength levels [32]. Recently, acute performance enhancements in CMJ [33,34,35], but not COD, following FEO have been reported, with larger inertial loads responsible for greater and faster improvements in some [33,34,35] but not all studies [36]. In addition, Wang et al. [37] evaluated the acute effects of different loads in FEO on drop jump performance. They reported that in the low-load condition (0.025 kg·m^2^), jump height decreased at 30 s (*p* = 0.01, d = 1.38) but increased significantly at 4 min (*p* < 0.05, d = 2.41), indicating that low-load eccentric overload successfully induced PAPE. When examining the time interval between conditioning activities and subsequent performance, most studies indicated that brief rest periods, approximately 3 min long, are effective in inducing PAPE [12]. However, periods identified as “immediately after” [29], as well as those lasting 1 min [30], 6 min [23], and 9 min [30], have also been documented. Those variations likely reflect dependance on the load of the conditioning activity [12], the specific performance outcome measures employed [29], and the strength level of participants [11].

Despite the generally reported benefits, the magnitude of performance improvements varied widely across aforementioned studies, ranging from trivial to large effects. Additionally, most of these studies involved participants from a spectrum of backgrounds, including nonathletes [28,29,30,33,34,36], young athletes [31], and university-level athletes [32]. Notably, only one study [37] incorporated professional, senior team sport athletes, which raises questions about the generalizability and efficiency of FEO-based PAPE protocols within elite athletic populations. Overall, it can be concluded that there is limited evidence about the acute effects of FEO on lower limb power performance and strength in elite team sport players. Moreover, no data are available regarding PAP time-course or the magnitude of the effects using a flywheel device in this specific population. Finally, no study to date has explored how FEO-based PAPE protocols impact eccentric performance metrics, although it has been reported that eccentric power metrics show a strong association with performance variables such as COD, sprint, and jump tests [38].

Thus, the primary objective of this study was to investigate the effects of PAPE induced by an FEO on CMJ performance metrics—specifically CMJH, CMJPP, and CMJRFDecc—as well as on COD and IMTP performance in elite team sport athletes. We hypothesize that the FEO-based PAPE protocol will produce significant acute improvements in CMJ performance metrics, as well as enhancements in COD and IMTP in this population.

## 2. Materials and Methods

### 2.1. Subjects

An a priori sample size estimation was performed using G*Power (version 3.1.9.3, Düsseldorf, Germany). Considering the study design—where participants were subjected to 4-time-point measurement—and utilizing a one-way ANOVA to assess the main effects of time with an assumed medium effect size (f = 0.35), an alpha level of 0.05, and a statistical power of 0.80, the analysis indicated that a minimum of 9 participants would be required. This sample size configuration yields an actual statistical power of 0.85. A total of 10 (3 female and 7 male athletes, age 28 ± 9 years, body weight 87.9 ± 20 kg, and body height 190 ± 17 cm) elite athletes participating in team sports (handball, basketball, football, and volleyball) were enrolled in the study. Specifically, five of the players are currently part of their nation’s official A national team (Serbia and Bosnia and Herzegovina), while the remaining five actively participate in the highest-quality European club competitions.

The measurement error (TEM) for both body height and body mass was below 0.02%. Height was assessed using a SECA measuring rod (Seca GmbH, Hamburg, Germany), which has a precision of 1 mm and a measurement range of 130–210 cm. Body mass was determined with a SECA model scale (accuracy of 0.1 kg; capacity of 2–130 kg). Participants were selected based on specific inclusion criteria: (I) no injuries in the past four months and no illnesses in the previous four weeks and (II) no involvement in club practices or individual sports training within two weeks prior to or during the study period. Prior to participation, all individuals received detailed information regarding the potential risks and benefits of the intervention and provided written informed consent. The study received approval from the University of Novi Sad’s ethics committee (Ref. No. 41-01-02/2024-1), and all procedures adhered to the principles outlined in the Declaration of Helsinki for research involving human subjects.

### 2.2. Experimental Approach to the Problem

**Figure 1:**
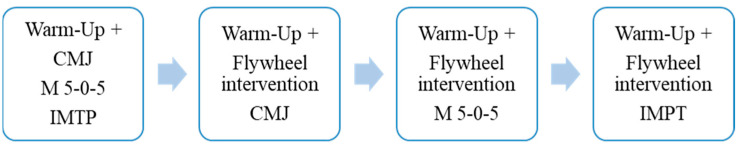
Flowchart of the study design and experimental procedures. CMJ—countermovement jump; M 5-0-5—modified 5-0-5 change-of-direction test; IMPT—isometric mid-thigh pull.

A randomized, crossover design was employed to assess the acute effects of eccentric overload exercise on countermovement jump (CMJ), change of direction (COD) ability, and isometric mid-thigh pull (IMTP) performance. Participants completed two familiarization sessions to acquaint themselves with baseline testing procedures. Baseline assessments, including CMJ and M505 IMTP, served as control measures (no post-activation potentiation [PAPE] stimulus) to evaluate the effects of the flywheel eccentric overload (FEO) protocol. One week later, participants started attending three additional testing sessions at 96 h intervals. Each session involved a standardized warm-up, flywheel conditioning activity, and one of three performance tests administered in randomized order immediately post-conditioning and after 3 and 6 min of passive recovery (see Figure 1 for layout). All tests were conducted during the 2024/2025 off-season, with sessions scheduled at the same time of day (morning) to mitigate circadian influences. Testing was performed by the same examiner (N.A., PhD student), supervised by a senior researcher (M.S.), to ensure consistency and data integrity. Participants completed procedures individually and were instructed to abstain from supplements and alcohol throughout the study. Given their routine visits to the Training Expertise Laboratory for regular testing and training, participants were familiar with the protocols, including the use of isokinetic devices, force platforms, and flywheel technology.

### 2.3. Intervention

Participants performed a general standardized warm-up procedure for each attended session. The warm-up protocol consisted of 5 min of cycling at constant power, followed by 7 min dynamic stretching and activation (bodyweight squats and bodyweight backward lunge), and finishing with ladder and landing skills, lasting around 15 min all together. A Flywheel kBox device (Exxentric, Bromma, Sweden) was used to perform the half squat with eccentric overload. The PAPE protocol consisted of three sets of six repetitions (with two initial repetitions for increase in the momentum of inertia), with 2 min passive recovery between sets. The moment of inertia was 0.025 kg·m^2^ and was the same for all participants.

The participants were instructed to exert maximum effort during the concentric phase, approaching near-full extension, and to continue seamlessly into the eccentric contraction phase without pause, delaying the lengthening of the braking phase until the final third of the eccentric phase to induce eccentric overload. Strong standardized encouragement was provided to ensure each repetition was fully performed.

### 2.4. Procedures

Countermovement Jump (CMJ) Test—The assessment of the CMJ was performed using a portable force platform (K-Deltas, Kinvent Inc., Montpellier, France). Participants began the assessment in an upright stance with their hands positioned on their hips throughout the procedure. Following a rapid downward movement into a self-selected semi-squat position, they performed a maximal vertical jump, ensuring landing with knees fully extended. Two attempts were permitted, and the highest recorded measurements for jump height, concentric peak power, and eccentric rate of force development (30 ms time intervals) were documented for further analysis.

Modified 5-0-5 change of direction test (COD)—For the m 5-0-5 test, photocells (Microgate—Witty, Italy) were used for timing. Participants began the test standing 0.3 m behind the starting gate. They performed a voluntary sprint covering 5 m, crossing the change-of-direction (COD) line with either foot, executing a 180-degree turn, and re-accelerating as rapidly as possible through the first timing gate. Each participant completed the test twice for right (COD5mR) and left (COD5mL) leg, and the best performance was selected for further evaluation.

Isometric Mid-Thigh Pull (IMTP)—Peak isometric force was evaluated during the IMTP using a portable force plate system (K-Deltas, Kinvent Inc., Montpellier, France). Participants initiated the test with their knee and hip angles set between 140 and 150 degrees to replicate the second phase of the clean. An immovable steel bar (TechnoGym, Cesena, Italy) was positioned at approximately mid-thigh level, just below the hip flexors. Each participant performed two trials, each lasting 5 s of maximal isometric effort, with a 60 s rest period between attempts. Participants received instructions and were strongly encouraged to exert maximum force as rapidly as possible during each trial, actively pushing their feet into the force plates. The highest force value recorded, measured in newtons, was used for subsequent analysis.

### 2.5. Statistical Analysis

Data analysis was conducted using IBM SPSS Statistics (version 29, IBM Corp., Armonk, NY, USA). The test–retest reliability of performance measures was evaluated using the intra-class correlation coefficient (ICC) with a two-way mixed-effects model for consistency, interpreted as follows: ≥0.9 indicates excellent reliability; 0.8–0.9 suggests good reliability; 0.7–0.8 is acceptable; 0.6–0.7 is questionable; 0.5–0.6 is poor; and below 0.5 is unacceptable. The assumption of sphericity was tested with Mauchly’s test. When violated, degrees of freedom were corrected with the Greenhouse–Geisser correction. A one-way repeated measures ANOVA was performed to examine the immediate effects of the flywheel eccentric overload protocol on performance parameters at baseline and at zero, three, and six minutes post-intervention.

Post hoc pairwise comparisons were calculated using the Least Significant Difference (LSD) test to explore significant main effects. Effect sizes were initially calculated using the partial eta-squared (η^2^) values, which were then converted to Cohen’s d using an online effect size conversion calculator (29). Effect sizes were interpreted by Cohen’s d as trivial (<0.2), small (≥0.2), moderate (≥0.6), large (≥1.2), and very large (>2.0) (11). For all statistical tests, a significance level of *p* < 0.05 was used.

## 3. Results

The conducted tests showed excellent test–retest reliability for CMJ height (ICC = 0.98, 95% CI: 0.94–0.99, TE = 0.82), CMJ PP (ICC = 0.99, 95% CI: 0.9–0.99, TE = 110.4), CMJ RFD_ecc_ (ICC = 0.93, 95% CI: 0.82–0.98, TE = 70.4), COD5mR (ICC = 0.92, 95% CI: 0.79.0.98, TE = 0.03), and IMTP (ICC = 0.98, 95% CI: 0.95–0.99, TE = 6.3), while CODL showed good test–retest reliability (ICC = 0.83, 95% CI: 0.54–0.95, TE = 0.05). The repeated measures ANOVA revealed significant effects for COD5mL (F = 8.38, *p* < 0.001, Cohen’s d = 1.92), COD5mR (F = 11.3, *p* < 0.001, Cohen’s d¬ = 2.24), CMJH (F = 12.4, *p* < 0.001, Cohen’s d = 2.35), and CMJPP (F = 6.4, *p* = 0.002, Cohen’s d = 1.68), while no overall statistically significant effects were reported for CMJRFDecc (*p* = 0.64) and IMTP (*p* = 0.5) (Table 1).

**Figure 2:**
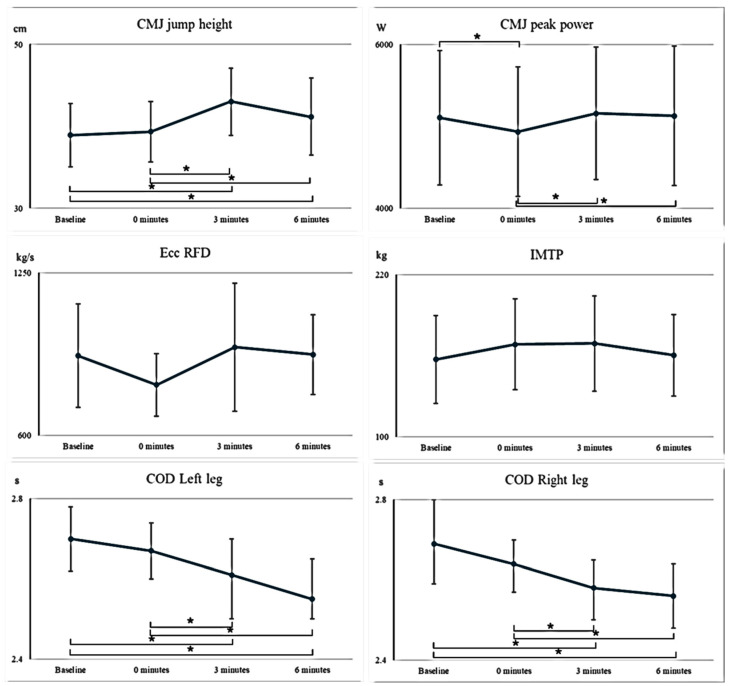
PAPE time window after a bout of flywheel training intervention. Data reported as mean ± 95% confidence interval. CMJ—countermovement jump; Ecc RFD—CMJ eccentric rate of force development; IMTP—isometric mid-thigh pull; COD—change of direction. * indicate significant difference between two time points.

For the COD5mL, pairwise comparisons revealed a statistically significant difference between the baseline assessment and the 3 min follow-up (*p* < 0.001, ES = 0.99). Additionally, significant differences were observed between the initial measurement and the 6 min assessment (*p* = 0.04, ES = 1.19), as well as between the 0 min and both the 3 min (*p* = 0.035, ES = 0.79) and 6 min assessments (*p* = 0.007, ES = 1.08) (Figure 2).

Pairwise comparisons for COD5mR showed similar results, with significant differences between the initial and 3 min measurements (*p* = 0.003, ES = 1.25), the initial and 6 min measurements (*p* < 0.001, ES = 1.09), the 0 min and 3 min measurements (*p* = 0.028, ES = 0.83), and the 0 min and 6 min measurements (*p* = 0.007, ES = 1.09).

Significant differences were also recorded for jump height between the initial and 3 min measurements (*p* < 0.001, ES= 1.62), initial and 6 min measurements (*p* = 0.01, ES = 1.02), the 0 min and 3 min measurements (*p* < 0.001, ES = 1.49), and the 0 min and 6 min measurements (*p* = 0.045, ES = 0.72). CMJPP showed a significant difference between the initial measurement and 0 min measurement (*p* = 0.024, ES = 0.85). In addition, there was a significant difference for CMJPP between the 0 min and 3 min measurements (*p* = 0.002, ES = 1.35), as well as between the 0 min and 6 min measurements (*p* = 0.018, ES = 0.91).

## 4. Discussion

To the authors’ knowledge, this study is the first to assess these parameters in this population after performing a squat exercise with a flywheel device. Compared to baseline measurements, a greater CMJ height was observed 3 and 6 min after the conditioning activity, as well as the period between immediately after the conditioning activity (0 min) and both the 3 and 6 min time points. CMJPP dropped significantly immediately after the conditioning activity but recovered to baseline values after 3 min. Both COD5mL and COD5mR showed significant improvements 3 and 6 min after the conditioning activity in comparison to baseline measurements, as well as between baseline and both 3 and 6 min. Finally, no overall statistically significant effects of FEO conditioning activity were reported for the CMJ eccentric rate of force development or isometric mid-thigh pull.

Numerous investigations have examined the acute influence of flywheel training interventions on CMJ performance, with the majority indicating a beneficial impact on various CMJ parameters. For example, Beato et al. [30] evaluated the effects of eccentric overload induced by flywheel devices on CMJ metrics at multiple time points—15 s and 1, 3, 5, 7, and 9 min after the conditioning activity—in a cohort of healthy adult males. Their findings demonstrated statistically significant enhancements, characterized by small to moderate effect sizes, in CMJ height at 3, 5, 7, and 9 min post-intervention, whereas no significant change was observed at the 15 s mark. These results are consistent with our observations, differing primarily in the magnitude of the effects. Contrary to our outcomes, they also identified modest improvements in peak power during CMJ at 1, 3, 5, 7, and 9 min but not at 15 s. These discrepancies may be attributable to sample size differences. Unlike the aforementioned studies, our research was conducted on elite athletes who were extensively familiarized with flywheel strength training, thereby likely requiring higher loads to induce acute improvements in power-related outcomes [39]. In line with our findings, Beato et al. [29] measured the effects of conditioning at thirty seconds, three minutes, and six minutes post-exercise, noting “very strong” to “extreme” improvements in CMJ height at three and six minutes following three sets of six maximal power flywheel half-squats in active male participants, while only anecdotal improvements were observed at thirty seconds. Also in active male participants, De Keijzer et al. [23] found positive post-activation potentiation effects on CMJ performance after similar conditioning, with significant effects at 6 min but not at 3 min. Shi et al. [36] demonstrated that light-load flywheel activities led to significant CMJ performance improvements at four, eight, and twelve minutes in non-athlete males. However, Fu et al. [33] showed no significant improvements at four minutes after a similar conditioning activity in active males. Notably, moderate and heavy flywheel resistances (0.078 and 0.156 kg·m^2^) appeared to enhance CMJ performance within four to twelve minutes post-exercise. Collectively, the aforementioned studies corroborate our study findings that flywheel load induces positive changes on CMJ height performance at 3 and 6 min after conditioning activity. Interestingly, flywheel eccentric overload did not significantly affect CMJRFDecc—the rate at which force is generated during eccentric (lengthening) muscle contractions—which could be expected based on flywheel training eccentric overload specificity and some previous study findings [37]. Furthermore, it has been documented that CMJRFDecc serves as a robust predictor of CMJ performance [40]. This is primarily because CMJRFDecc encapsulates multiple intrinsic properties of muscle and tendons during a braking phase, which significantly influences performance outcomes. Given the absence of the observed effects of flywheel conditioning activity on CMJRFDecc, it is plausible to hypothesize that the observed improvements in CMJ height are primarily attributable to short-term neural responses, including increased alpha motor neuron excitability and synchronized motor unit recruitment [2]. These combined neural mechanisms may facilitate more efficient cross-bridge cycling and heightened muscle fiber activation, thereby contributing to the enhancement of CMJ performance.

Flywheel training has been widely documented to induce long-term improvements in change of direction (COD) ability, primarily through its capacity to enhance eccentric strength, concentric power, and neuromuscular control [41]. In fact, a single weekly session of flywheel parallel squats (inertia: 0.11 kg·m·s^2^) conducted over a 10-week period has been shown to produce significantly greater gains (moderate-to-large effect size) in COD performance compared to a group performing the same exercise with traditional loading methods (i.e., 80% 1-RM) in conjunction with routine soccer-specific training [42]. In addition, few studies have investigated the acute effects of flywheel conditioning activity on change of direction ability. Beato et al. 2021 [29] reported “extreme” and “strong” Bayesian probabilities for improvement at 3 and 6 min, as well as a “strong” probability for improvement 6 min following medium- and high-load flywheel conditioning activity. This is aligned with our results that show a significant improvement 3 and 6 min following low-load flywheel conditioning activity. Similar findings were presented by Beato et al. [43], who reported significant improvements at 4 min post-flywheel eccentric overload in three groups: cross-cutting step with conical pulley, knee extension, and squat exercises. Somewhat contrasting to our study findings, McErlain-Naylor and Beato [34] reported that a flywheel half-squat protocol (3 × 6 repetitions with 0.029 kg·m^2^) did not elicit a PAPE effect on resulting ground reaction force parameters during COD movements in recreational team sport athletes. However, since COD times were not reported, the findings from this study should be considered circumstantial and interpreted with caution. Consistent with this, our results also indicate that some key ground reaction force parameters (e.g., RFDecc) were unaffected by the conditioning activity, whereas a significant improvement in CMJ was observed. In addition, a more recent study [44] found no effect of flywheel on change of direction ability, most likely due to only one set of flywheel eccentric overload being implemented, which has been suggested to be insufficient for achieving PAPE [23]. Collectively, the study’s findings suggest generally positive effects of higher-volume flywheel conditioning activity on subsequent change of direction performance, which is in accordance with our study’s findings.

To the best of the authors’ knowledge, this is the first study to examine the acute effects of flywheel eccentric overload on the isometric mid-thigh pull (IMTP) strength test. The results show non-significant increases at all three time points (0 s, 3 min, and 6 min post-conditioning activity). Recently, Tseng et al. [6] reported that three sets of four repetitions of eccentric overload half-squats (eccentric: 105% of concentric 1RM; concentric: 80% of concentric 1RM) produced a non-significant decrease in IMTP peak performance 10 min after conditioning. Considering the similar overall training load between the studies (3 × 6 vs. 3 × 4 repetitions), it is plausible that differences in outcomes may be attributed to the participants’ strength levels. Elite athletes, such as those in our study, likely possess higher strength levels, which may confer greater resistance to fatigue and mitigate the negative effects of fatigue on subsequent maximal strength performance [34]. Several other studies have investigated the acute strength response to flywheel conditioning activities. For example, Beato et al. [30] reported trivial (ES = 0.13, *p* = 0.001) acute improvements in the isokinetic concentric peak torque of the quadriceps and hamstring following flywheel interventions, with effects observed from 3 to 9 min post-conditioning. In another study, Beato et al. [20] found a small non-significant effect (ES = 0.31, *p* = 0.233) for isokinetic quadriceps concentric peak torque at 5 min following flywheel half-squat conditioning. The authors suggest that the limited PAPE effect of flywheel conditioning on isokinetic quadriceps peak torque may be related to kinematic differences between the exercises (closed kinetic chain vs. open kinetic chain) used in the pre-load exercise and the subsequent test. Overall, the effects of flywheel conditioning activity on subsequent strength performance appear to be small or nonexistent, regardless of whether strength was measured with open or closed kinetic chain modalities, as in our study. While further research is warranted, it can be concluded that using flywheel exercises to induce acute strength improvements does not seem to be a viable strategy for enhancing maximal strength performance in the sample of elite team sport athletes.

The primary limitation of this study is the relatively small sample size with only ten athletes of mixed sex, which could limit the generalizability of the findings. Second, the investigation employed a single protocol with a moment of inertia of 0.025 kg·m^2^, creating uneven relative loading and likely influencing the magnitude of PAPE response. Future research should explore various inertial loads to yield more precise insights into the potentiation of PAPE effects associated with flywheel squat exercises on performance metrics. Finally, to better understand the temporal aspects of PAPE, future studies should monitor longer post-activation periods to observe how performance outcomes change over time.

## 5. Conclusions

In conclusion, this study shows that FEO exercise can increase CMJ and COD performance in an elite team sport population. PAPE onset was found at three minutes with large and moderate effect sizes, respectively, while performance gains were affected immediately after the exercise. In addition, no significant changes were observed for CMJRFDecc, while a significant drop was reported for CMJPP immediately after conditioning activity, but baseline values were regained at the three-minute time point. This study has not found a meaningful PAPE effect of FEO on maximum strength levels (isometric mid-thigh pull performance). It seems that this PAPE-induced modality may be used to acutely enhance horizontal and vertical power performance but not maximum strength levels in this specific cohort.

### Practical Applications

The current results may assist coaches in enhancing strength and power progression during training phases (such as contrast training) and prior to competitions that demand high levels of power and strength. These study’s findings suggest that a flywheel overload conditioning activity involving three sets of six reps with a medium moment of inertia (0.025 kg m^2^) might induce transient enhancements in vertical jump height and change of direction performance in elite team sport athletes, particularly three and six minutes following exercise. However, since no immediate effects were observed post-exercise, the timing of subsequent explosive actions should be carefully scheduled, preferably allowing a recovery period of three minutes. Additionally, given the lack of significant alterations in isometric mid-thigh pull performance, reliance on flywheel exercises for acute strength gains may be limited.

## Figures and Tables

**Table 1:** CMJ—countermovement jump; CMJPP—countermovement jump peak power; CMJRFDecc—countermovement jump eccentric rate of force development; COD5mL—change of direction 5 m left leg; COD5mR—change of direction 5 m right leg; IMPT—isometric mid-thigh pull.

	Baseline Testing		0 Post-Intervention		3 min Post-Intervention		6 min Post-Intervention		F	Sign.	Cohen’s d
	Mean ± SD	CI 95%	Mean ± SD	CI 95%	Mean ± SD	CI 95%	Mean ± SD	CI 95%			
CMJH (cm)	38.92 ± 5.43	[35.03, 42.8]	39.34 ± 5.16	[35.65, 43.0]	43.02 ± 5.75	[38.9, 47.14]	41.16 ± 6.56	[36.5, 45.9]	12.44	<0.001	2.35
CMJ PP (W)	5106.90 ± 1150.57	[4283.8, 5929.9]	4935.90 ± 1106.85	[4144.1, 5727.7]	5160.30 ± 1131.54	[4350.8, 5969.8]	5129.10 ± 1190.87	[4277.2, 5981.0]	6.40	<0.002	1.68
CMJ RFDecc (N/s)	9011.33 ± 2830.68	[6986.26, 11,036.40]	7864.93 ± 1718.22	[6636.16, 9093.70]	9342.79 ± 3511.76	[6830.33, 11,855.25]	9053.49 ± 2187.66	[763.6, 7488.35]	2.71	>0.64	
COD5mL (s)	2.70 ± 0.11	[2.62, 2.78]	2.67 ± 0.08	[2.6, 2.7]	2.61 ± 0.09	[2.54, 2.68]	2.55 ± 0.13	[2.45, 2.65]	8.30	<0.001	1.92
COD5mR (s)	2.69 ± 0.13	[2.6, 2.8]	2.64 ± 0.09	[2.57, 2.7]	2.58 ± 0.10	[2.5, 2.65]	2.56 ± 0.11	[2.5, 2.64]	11.311	<0.001	2.24
IMTP (N)	1543.57 ± 446.59	[1223.87, 1862.28]	1651.54 ± 461.40	[1321.93, 1981.92]	1659.77 ± 484.05	[1313.11, 2006.44]	1573.28 ± 414.62	[1276.82, 1870.13]	2.229	>0.5	

## Data Availability

Data are available upon reasonable request.

## References

[1] Blazevich A.J., Babault N. (2019). Post-activation potentiation versus post-activation performance enhancement in humans: Historical perspective, underlying mechanisms, and current issues. Front. Physiol..

[2] Tillin N.A., Bishop D. (2009). Factors modulating post-activation potentiation and its effect on performance of subsequent explosive activities. Sports Med..

[3] Seitz L.B., Haff G.G. (2015). Factors modulating post-activation potentiation of jump, sprint, throw, and upper-body ballistic performances: A systematic review with meta-analysis. Sports Med..

[4] Chu D.A. (1996). Explosive Power & Strength: Complex Training for Maximum Results. http://ci.nii.ac.jp/ncid/BA28426942.

[5] Stone M.H., Sands W.A., Pierce K.C., Ramsey M.W., Haff G.G. (2008). Power and power potentiation among strength–power athletes: Preliminary study. Int. J. Sports Physiol. Perform..

[6] Tseng K., Chen J., Chow J., Tseng W., Condello G., Tai H., Fu S. (2021). Post-activation performance enhancement after a bout of accentuated eccentric loading in collegiate male volleyball players. Int. J. Environ. Res. Public Health.

[7] Bauer P., Sansone P., Mitter B., Makivic B., Seitz L.B., Tschan H. (2018). Acute effects of back squats on countermovement jump performance across multiple sets of a contrast training protocol in resistance-trained men. J. Strength Cond. Res..

[8] Iacono A.D., Martone D., Padulo J. (2016). Acute effects of drop-jump protocols on explosive performances of elite handball players. J. Strength Cond. Res..

[9] Hodgson M., Docherty D., Robbins D. (2005). Post-activation potentiation. Sports Med..

[10] Rassier D., MacIntosh B. (2000). Coexistence of potentiation and fatigue in skeletal muscle. Braz. J. Med. Biol. Res..

[11] Seitz L.B., Trajano G.S., Haff G.G. (2014). The back squat and the power clean: Elicitation of different degrees of potentiation. Int. J. Sports Physiol. Perform..

[12] Beato M., McErlain-Naylor S.A., Halperin I., Dello Iacono A. (2019). Current evidence and practical applications of flywheel eccentric overload exercises as postactivation potentiation protocols: A brief review. Int. J. Sports Physiol. Perform..

[13] Wallace B.J., Shapiro R., Wallace K.L., Abel M.G., Symons T.B. (2019). Muscular and neural contributions to postactivation potentiation. J. Strength Cond. Res..

[14] French D.N., Kraemer W.J., Cooke C.B. (2003). Changes in dynamic exercise performance following a sequence of preconditioning isometric muscle actions. J. Strength Cond. Res..

[15] Güllich A., Schmidtbleicher D. (1996). MVC-induced short-term potentiation of explosive force. New Stud. Athl..

[16] Behm D.G., Button D.C., Barbour G., Butt J.C., Young W.B. (2004). Conflicting effects of fatigue and potentiation on voluntary force. J. Strength Cond. Res..

[17] Gossen E.R., Sale D.G. (2000). Effect of postactivation potentiation on dynamic knee extension performance. Eur. J. Appl. Physiol..

[18] De Villarreal E.S.S., González-Badillo J.J., Izquierdo M. (2007). Optimal warm-up stimuli of muscle activation to enhance short- and long-term acute jumping performance. Eur. J. Appl. Physiol..

[19] Gourgoulis V., Aggeloussis N., Kasimatis P., Mavromatis G., Garas A. (2003). Effect of a submaximal half-squats warm-up program on vertical jumping ability. J. Strength Cond. Res..

[20] Rahimi R. (2007). The acute effects of heavy versus light-load squats on sprint performance. Facta Univ. Ser. Phys. Educ. Sport.

[21] Bazett-Jones D.M., Winchester J.B., McBride J.M. (2005). Effect of potentiation and stretching on maximal force, rate of force development, and range of motion. J. Strength Cond. Res..

[22] Moir G.L., Dale J.R., Dietrich W.W. (2009). The acute effects of heavy back squats on mechanical variables during a series of bilateral hops. J. Strength Cond. Res..

[23] De Keijzer K.L., McErlain-Naylor S.A., Dello Iacono A., Beato M. (2020). Effect of volume on eccentric overload–induced postactivation potentiation of jumps. Int. J. Sports Physiol. Perform..

[24] Maroto-Izquierdo S., García-López D., Fernandez-Gonzalo R., Moreira O.C., González-Gallego J., De Paz J.A. (2017). Skeletal muscle functional and structural adaptations after eccentric overload flywheel resistance training: A systematic review and meta-analysis. J. Sci. Med. Sport.

[25] Núñez F.J., Suarez-Arrones L.J., Cater P., Mendez-Villanueva A. (2016). The high-pull exercise: A comparison between a VersaPulley flywheel device and free weights. Int. J. Sports Physiol. Perform..

[26] Cormie P., McGuigan M.R., Newton R.U. (2011). Developing maximal neuromuscular power. Sports Med..

[27] Chiu L.Z., Barnes J.L. (2003). The fitness-fatigue model revisited: Implications for planning short- and long-term training. Strength Cond. J..

[28] Timon R., Allemano S., Camacho-Cardeñosa M., Camacho-Cardeñosa A., Martinez-Guardado I., Olcina G. (2019). Post-activation potentiation on squat jump following two different protocols: Traditional vs. inertial flywheel. J. Hum. Kinet..

[29] Beato M., De Keijzer K.L., Leskauskas Z., Allen W.J., Dello Iacono A., McErlain-Naylor S.A. (2021). Effect of postactivation potentiation after medium vs. high inertia eccentric overload exercise on standing long jump, countermovement jump, and change of direction performance. J. Strength Cond. Res..

[30] Beato M., Stiff A., Coratella G. (2019). Effects of postactivation potentiation after an eccentric overload bout on countermovement jump and lower-limb muscle strength. J. Strength Cond. Res..

[31] De Hoyo M., De La Torre A., Pradas F., Sañudo B., Carrasco L., Mateo-Cortes J., Domínguez-Cobo S., Fernandes O., Gonzalo-Skok O. (2014). Effects of eccentric overload bout on change of direction and performance in soccer players. Int. J. Sports Med..

[32] Beato M., De Keijzer K.L., Fleming A., Coates A., La Spina O., Coratella G., McErlain-Naylor S.A. (2020). Post flywheel squat vs. flywheel deadlift potentiation of lower limb isokinetic peak torques in male athletes. Sports Biomech..

[33] Fu K., Chen L., Poon E.T., Wang R., Li Q., Liu H., Ho I.M.K. (2023). Post-activation performance enhancement of flywheel training on lower limb explosive power performance. Front. Physiol..

[34] McErlain-Naylor S.A., Beato M. (2021). Post flywheel squat potentiation of vertical and horizontal ground reaction force parameters during jumps and changes of direction. Sports.

[35] Shi J., Yan B., Yu M., Wang Z., Wang Y., Liu H., Zhang W., Girard O. (2024). Heavier loads in flywheel exercise induce greater post-activation performance enhancement in countermovement jumps compared to heavy Smith machine squats in males. Biol. Sport.

[36] Tsoukos A., Tsoukala M., Papadimitriou D.M., Terzis G., Bogdanis G.C. (2025). Acute effects of low vs. high inertia during flywheel deadlifts with equal-force impulse on vertical jump performance. Sensors.

[37] Wang X., Zhai H., Wei H. (2025). Acute effects of different intensities of flywheel half squat based on velocity on vertical jump performance in high-level athletes. Appl. Sci..

[38] Kaya S., Ersöz M. (2025). Performance predictors in elite athletes: Evaluating the role of eccentric utilization ratio and mechanical power outputs. Appl. Sci..

[39] Seitz L.B., De Villarreal E.S., Haff G.G. (2013). The temporal profile of postactivation potentiation is related to strength level. J. Strength Cond. Res..

[40] Laffaye G., Wagner P. (2013). Eccentric rate of force development determines jumping performance. Comput. Methods Biomech. Biomed. Eng..

[41] Beato M., De Keijzer K.L., Muñoz-Lopez A., Raya-González J., Pozzo M., Alkner B.A., Dello Iacono A., Vicens-Bordas J., Coratella G., Maroto-Izquierdo S. (2024). Current guidelines for the implementation of flywheel resistance training technology in sports: A consensus statement. Sports Med..

[42] Coratella G., Beato M., Cè E., Scurati R., Milanese C., Schena F., Esposito F. (2019). Effects of in-season enhanced negative work-based vs. traditional weight training on change of direction and hamstrings-to-quadriceps ratio in soccer players. Biol. Sport.

[43] Beato M., Madruga-Parera M., Piqueras-Sanchiz F., Moreno-Pérez V., Romero-Rodriguez D. (2019). Acute effect of eccentric overload exercises on change of direction performance and lower-limb muscle contractile function. J. Strength Cond. Res..

[44] Loturco I., Pereira L., Zabaloy S., Mercer V., Moura T.B.M.A., Freitas T.T., Boullosa D. (2024). No post-activation performance enhancement following a single set of plyometric or flywheel exercises in national team rugby players. Appl. Sci..

